# Digital Traces of Behaviour Within Addiction: Response to Griffiths (2017)

**DOI:** 10.1007/s11469-017-9855-7

**Published:** 2018-01-03

**Authors:** David A. Ellis, Linda K. Kaye, Thomas D.W. Wilcockson, Francesca C. Ryding

**Affiliations:** 1 Lancaster University, Lancaster, UK; 2 Department of Psychology, Edge Hill University, St Helens Road, Ormskirk, Lancashire L39 4QP UK

**Keywords:** Internet addiction, Internet gaming disorder, Smartphone addiction, Behavioural tracking, Digital traces

## Abstract

Griffiths’ (2017) response to the recent commentary piece by Ryding and Kaye (2017) on “Internet Addiction: A conceptual minefield” provided a useful critique and extension of some key issues. We take this opportunity to further build upon on one of these issues to provide some further insight into how the field of “internet addiction” (IA) or technological addictions more generally, may benefit from capitalising on behavioural data. As such, this response extends Griffiths’ (2007) points surrounding the efficacy of behavioural data previously used in studies on problematic gambling, to consider its merit for future research on IA or associated topics such as Internet Gaming Disorder (IGD) or “Smartphone addiction”. Within this, we highlight the challenges associated with utilising behavioural data but provide some practical solutions which may support researchers and practitioners in this field. These recent developments could, in turn, advance our understanding and potentially validate such concepts by establishing behavioural correlates, conditions and contexts. Indeed, corroborating behavioural metrics alongside self-report measures presents a key opportunity if scholars and practitioners are to move the field forward.

The recent commentary piece by Ryding and Kaye (2017) makes reference to the conceptual issues surrounding “Internet addiction” (IA). In response to this, Griffiths (2017) provided a useful critique and extension of this, in which he referred to a number of key issues. One of these relates to the utility of behavioural data which has been found to be beneficial in studies on problematic gambling (Auer & Griffiths, 2013, 2014, 2015, 2017; Braveman & Shaffer 2012). For example, some disciplines have used digital life-log images via a wearable device to capture different types of travel behaviour (Kelly, Doherty, Berry, Hodges, Batterham, & Foster 2011). In commercial settings, it is also worth noting that consumer behaviours (e.g. selecting content on websites) are used for commercial gain and to bolster advertising effectiveness (Robinson, Wysocka & Hand 2015). Indeed, we support this notion and acknowledge this as a fruitful endeavour in the realm of IA and other topics such as Internet Gaming Disorder (IGD) and “Smartphone addiction”. Specifically, technologies themselves now allow researchers to access and quantify behaviour at a level which was previously impossible. This includes a variety of behavioural markers (or digital traces) from social media, Smartphones and other wearable devices (Miller 2012; Piwek, Ellis & Andrews 2016a; Youyou, Kosinski & Stillwell, 2015). These can provide behavioural indicators (e.g. usage, movement) and contextual markers (e.g. location, time) upon which to form future empirical enquiries. However, very few papers utilise these digital traces of behaviour and typically rely on self-report alone. Although Griffiths (2017) does provide a very useful summary of how research into problematic gambling has utilised types of behavioural data, a frank discussion regarding similar issues of measurement would help the field move forward more quickly, improve its visibility and generate additional impact from a policy and practitioner perspective.

Approaches to using behavioural data range in complexity, from developing stand-alone software to making the most of existing application programming interfaces (APIs). Both approaches require some computer coding skills, but many applications already exist that can collect and analyse data within an easy to use graphical environment (e.g. http://chorusanalytics.co.uk/, http://socialdatalab.net/software). However, while these tools are being utilised in other disciplines such as computer science, there is an apparent absence within psychology as a whole, which is regrettable for a field that continues to drive theoretical developments forward. Instead, research has continued to rely on self-report measures to quantify and measure interactions with technology. For example, in the case of “Smartphone addiction”, particularly as this concept is gaining momentum in the literature (Kwon, Lee, Won, Park, Min, Hahn et al. 2013; Samaha & Hawi, 2016), not a single “Smartphone addiction” or problematic use scale has been validated against real-world Smartphone usage behaviours (De Sola Gutierrez, Rodriguez de Fonseca, Rubio 2016). At the same time, research in other disciplines has operationalized usage logging systems via Smartphones as a means by which to explore these issues, although due to a lack of psychological input, these have yet to corroborate with validated psychometric instruments (Ahn, Wijaya, & Esmero 2014). Further, research that has compared self-reported Smartphone behaviours with digital traces has found discrepancies between these two approaches (Andrews, Ellis, Shaw & Piwek 2015). Namely, Smartphone users are unable to recall the number of times they check their Smartphone on a daily basis (Andrews et al. 2015). This raises the question that existing measures of self-report are not always suitable when capturing related unconscious behaviours (Stacy 1997), which makes it difficult to advance conceptual understanding surrounding problematic use or impulsive behaviours, and thus contribute to debates related to online addictions. Specifically, the cognitive processes associated with compulsive use would conceivably be automatic (Stacy 1997) and as such, could not be captured adequately through self-reports which may only measure deliberate, conscious behaviours. Rather self-reports may, at best only be measuring expectancies associated with a given problematic behaviour. Further self-reports may be bound by judgement-memory relationships which may interfere with perceptions of one’s behaviour (Feldman & Lynch 1988; Wyer & Srull 1989). As such, someone who completes a self-report regarding positive outcomes regarding Smartphone use may readily, but not correctly make inferences such as “I use my Smartphone a lot, so therefore it must be pleasant for me”. Here, there may be the potential for using implicit association measures to identify implicit cognitive processes associated with problematic Smartphone use, for example (Wiers & Stacy 2006). This may help establish the extent to which implicit cognitions associated with Smartphones (or indeed other technological addictions) may mirror equivalent processes to that of other established substance use or behavioural addictions.

Beyond these research paradigms, it also makes conceptual and practical sense to draw on users’ actual behaviour to help resolve this issue. Such behaviour may be obtained through inbuilt apps within Smartphones which can run in the background and may capture checking and usage behaviours and how these may vary across time. As such, these may provide further validation of existing self-report problematic use scales to establish behavioural correlates of diagnostic criteria. Although this approach can primarily contribute to debates surrounding “Smartphone addiction” (i.e. to the device itself), it may also be useful to consider the potential for such apps to monitor behaviours within certain Internet environments which may be accessed via mobile devices. As such, these may obtain information about usage patterns on particularly Internet domains and thus provide objective data in respect of sub-types of IA or problematic use.

Smartphone applications can also capture contextual information automatically via the many on-board sensors (e.g. location, ambient light levels; Piwek & Ellis 2016). That is, this may address the issues raised both by Ryding and Kaye (2017) and noted by Griffiths (2017), regarding how context is important to note in this area of research. Namely, garnering usage data in correspondence with location data may provide additional insight into the contextual affordances associated with certain types of usage. Gaining objective data on this would extend the small-scale findings of Fullwood, Quinn, Kaye and Redding (2017) who, in respect of Smartphone usage itself, report that users often vary their Smartphone behaviours based on context. However, larger-scale research of this nature is lacking, and calls for further consideration of building research paradigms which may account of contextual variations when exploring Smartphone usage or mobile-enabled Internet behaviours.

Beyond concerns relating to Smartphones, behavioural data may also be garnered in respect of online gaming so as to further substantiate debate on IGD (Griffiths, Van Rooij, Kardefelt-Winther, Starcevic, Kiraly, Pallesen, et al. 2016; Petry, Rehbein, Gentile, Lemmens, Rumpf, Mossle, et al. 2014). This may involve log-in periods through game software, for example. It is interesting to note however, that other aspects of research outside the addiction field have utilised the online game environment from which to obtain performance data as a measure of skill acquisition (Stafford & Dewar 2014). Regrettably, this is the exception rather than the norm in psychological research and seems to remain aspirational rather than attainable in the context of the current trends in research. It is worth noting that research papers which generate significant impact have gone beyond self-report (Wagner, Barnes, Lim, & Ferris 2012; Youyou, Kosinski & Stillwell, 2015). Intriguingly, this does not appear to be happening within the field of cyberpsychology as a whole (Howard & Jayne, 2015), which is one sub-discipline which one may expect to be at the forefront of these advances. While it remains difficult to pinpoint the exact reasons why this trend exists a number of potential avenues are worthy of further discussion.

## Value Placed on Self-Report Measures

There remains a general consensus by psychologists that while traditional psychometric measures are far from perfect, they are the best option currently available. As a result, psychological research continues to place enormous value on traditional self-report based tests for a number of reasons. First, they are straightforward to administer and score. Second, they allow for norms to be calculated so individuals can be compared and contrasted on specific constructs. Third, they comprise a key part of psychological research training and so are familiar to researchers. Finally, the data is easy to interpret by non-experts making them ideal in an applied context (Kagan 2001; Lewis 2001). As such, psychology researchers may be prone to selecting self-reports because they remain familiar and convenient.

In contrast, objective behaviour including signals derived from digital traces is less straightforward to administer or interpret. However, there are many instances in which behavioural data is highly appropriate and, importantly, relatively attainable to be used as part of research design. In the case of Smartphone usage, for example, self-report measures have yet to be validated against any strong behavioural measure and any estimate remains problematic, yet this remains a relatively “easy” example of how behavioural usage metrics may be garnered and utilised in psychometric enquiry (Andrews et al. 2015). Employing objective measures of digital behaviour is also likely to help challenge and drive the development of existing psychometric tests, which may require significant modification as existing instruments are likely to be weak when it comes to predicting any real-world behaviour (e.g. screen time) when measured by a digital trace. Indeed, this is particularly pertinent given that many scales within the area of IA, IGD and “Smartphone addiction” have not yet been adequately tested in this regard, in terms of their validity and psychometric properties.

Of course, there will be many occasions where pen and paper assessments will be vital to a successful research design and traditional psychometric measures are frequently selected due to the fact that adequate training is provided as part of researcher training. They are therefore likely to be deemed a safe and familiar option as research tools. Correspondingly, the subjective self-report approach is historically embedded within the psychology, which means any change to shift such a long-standing and established paradigm is likely to be met with some resistance (Wagner et al. 2012). Although behavioural data is routinely collected during experiments, it becomes harder to quantify as part of observational research paradigms and is often dismissed due to practical demands of conducting research “in the field” (Boice 1983). It is probable that such a “stigma” upon observational or behavioural research still prevails, which may also go some way to explain the apparent reluctance to capitalise on these now more contemporary forms of behavioural data.

## Awareness of Methodological Advances in the Field

Beyond issues associated with self-report, many researchers may simply be unaware of the tools and methods at their disposal when it comes to directly measuring digital traces, perhaps because this is rarely covered as part of standard psychological training. This may also be, in part, the fault of those who develop new methods in the first instance because they often lack documentation that will enable novice users to adopt them as part of their research. These issues relate to both the collection and analysis of subsequent data. However, this situation may be improving particularly with regard to experience sampling (Thai & Page-Gould 2017). In addition, there are a growing number of freely available resources and this even includes many pre-built applications for Smartphones and social media platforms that will provide access to key digital metrics (see Piwek & Ellis 2016).

However, even if researchers are aware of the tools available, the resources required to collect and interpret digital behaviour will always be greater than administering a survey, but this can lead to gains elsewhere as data intensive designs can often rely on smaller sample sizes, particularly if the resolution of data from each participant is particularly high. Unfortunately, the pressure to publish quickly and frequently means that this extra time is a luxury many researchers are unable to afford (Sarewitz 2016). Similar problems also filter down into the process of peer review. As the speed of methodological development increases, reviewers are in a constant battle to keep up with these developments, which for most is impossible given other academic commitments. This may mean that many feel they are unable to review such papers or feel that they are unsuitable for publication because new methods have yet to be validated. Indeed, more work is often required when it comes to validating a new measure of behaviour when compared with the validation of a new measure that involves self-report alone, which will typically only involve exploratory and confirmatory factor analysis (Wagner et al. 2012). This is where more interdisciplinary work is essential when it comes to developing new methods that both researchers and reviewers are able to understand.

## Conclusion

Griffiths (2017) provided a useful critique and extension of Ryding and Kaye’s (2017) commentary on “Internet addiction: A conceptual minefield”. Here, he made reference to the utility of behavioural data to supplement this field. This response extends the discussion further and highlights the benefits and challenges of this approach within the field of IA and associated topics of IGD and “Smartphone addiction”. It seems there remains a large methodological gap when it comes to measuring and understanding digital traces which may be associated with aspects of problematic use in respect of Smartphones themselves or indeed specific online activities. In terms of capitalising on digital traces as they exist today, we would argue that the benefits of engaging with these new methods far outweigh the negatives particularly in terms of the additional impact that could be generated by any subsequent research. For more technically demanding projects, embracing and seeking-out interdisciplinary collaborations with colleagues in computer science and related fields (e.g. data science) will bring a multitude of benefits (Rhoten & Parker 2004), particularly if the objective is to develop interventions around behavioural change (Piwek, Ellis, Andrews & Joinson, 2016b). Finally, while our assessment of existing methodological approaches is less than positive, we strongly believe that researchers are in a strong position to capitalise on recent methodological developments as they relate to digital traces as they have much to offer, including the ability to build new theoretical frameworks around existing and future methodological developments. In turn, research that aims to conceptualise IA, IGD and “Smartphone addiction” could become the first to provide clarity when it comes to defining how nuanced behaviours associated with different Internet activities might align themselves with diagnostic criteria and associated psychological constructs.
